# Forward-viewing echoendoscope aids tissue acquisition via the afferent limb after pancreaticoduodenectomy

**DOI:** 10.1055/a-2302-9657

**Published:** 2024-04-29

**Authors:** Soma Fukuda, Susumu Hijioka, Yoshikuni Nagashio, Yuta Maruki, Mark Chatto, Yutaka Saito, Takuji Okusaka

**Affiliations:** 1 13874 Department of Hepatobiliary and Pancreatic Oncology, National Cancer Center Japan, Tokyo, Japan; 2 37571 Department of Medicine, Makati Medical Center, Manila, Philippines; 3 68380 Endoscopy Division, National Cancer Center Hospital, Tokyo, Japan

Endoscopic ultrasound-guided tissue acquisition (EUS-TA), commonly performed with an oblique-viewing echoendoscope, can be difficult in patients with surgically altered anatomy 1 . Recently, EUS-TA using an oblique-viewing echoendoscope inserted over a guidewire into the afferent limb has been reported 2 , but there is the risk of perforation. Although forward-viewing echoendoscopes can be safely inserted into the distal intestinal tract, there are few reports about EUS-TA via the afferent limb using them 3 4 . Here, we describe a patient with surgically altered anatomy who underwent EUS-TA using a forward-viewing echoendoscope for recurrent cancer of the distal bile duct.

The 85-year-old man had previously undergone pancreaticoduodenectomy with modified Child’s reconstruction for distal bile duct cancer. Two years later, computed tomography revealed a 30-mm intra-abdominal mass behind the portal vein ( Fig. 1 ), suggestive of bile duct cancer recurrence. We attempted EUS-TA using a transgastric approach. However, the mass puncture could not be performed because of the intervening portal vein ( Fig. 2 ). Therefore, a decision was made to perform EUS-TA via the afferent limb using a forward-viewing echoendoscope (TGF-UC260J; Olympus, Tokyo, Japan) instead ( Fig. 3 **a** , **b** ). The colonoscope was inserted into the afferent limb, followed by a guidewire, and the colonoscope was removed. Next, the echoendoscope was inserted into the afferent limb over the guidewire under fluoroscopic guidance and endoscopic vision ( Fig. 3 **c** ). EUS successfully showed a hypoechoic mass adjacent to the portal vein ( Fig. 4 **a** , **b** ). EUS-TA was performed without complications using a 22-gauge Franseen needle ( Fig. 4 **c** , **d** , Video 1 ). The histopathological diagnosis was adenocarcinoma, consistent with bile duct cancer recurrence ( Fig. 5 ).

**Fig. 1:**
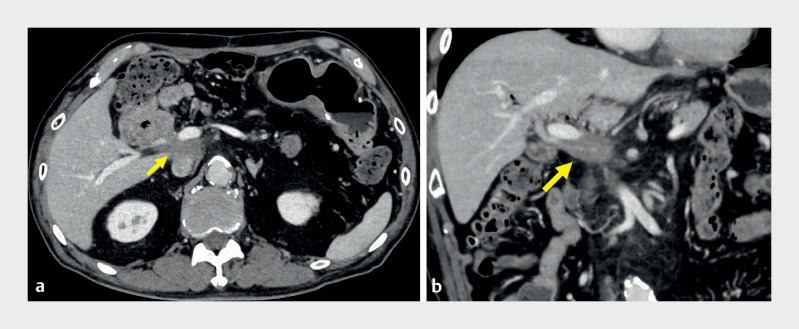
Contrast-enhanced computed tomography showing a 30-mm hypovascular mass (arrow) behind the portal vein. **a** Axial image. **b** Coronal image.

**Fig. 2:**
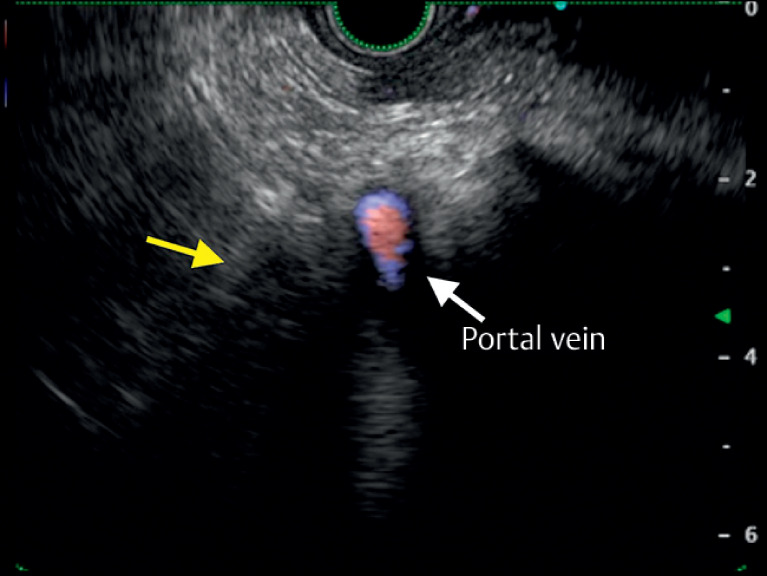
Transgastric echoendoscopic image showing the obscure mass (arrow) with the intervening portal vein.

**Fig. 3:**
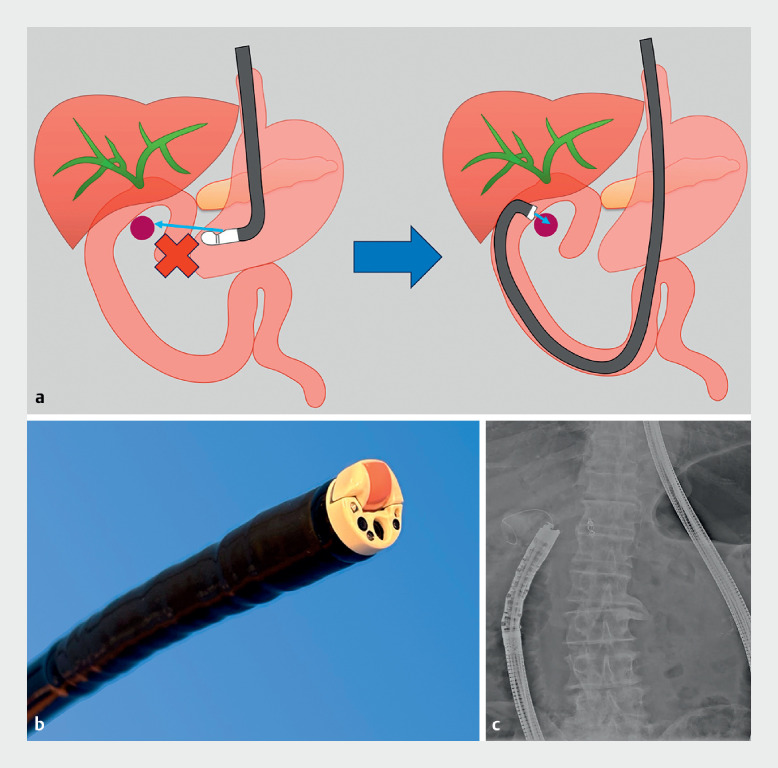
**a** Endoscopic ultrasound-guided tissue acquisition (EUS-TA) with an oblique-viewing echoendoscope was technically unfeasible due to positional difficulty. Hence, a decision was made to perform EUS-TA via the afferent limb using a forward-viewing echoendoscope instead. **b** Forward-viewing echoendoscope (TGF-UC260J; Olympus, Tokyo, Japan). **c** Fluoroscopic image showing the forward-viewing echoendoscope inserted into the afferent limb.

**Fig. 4:**
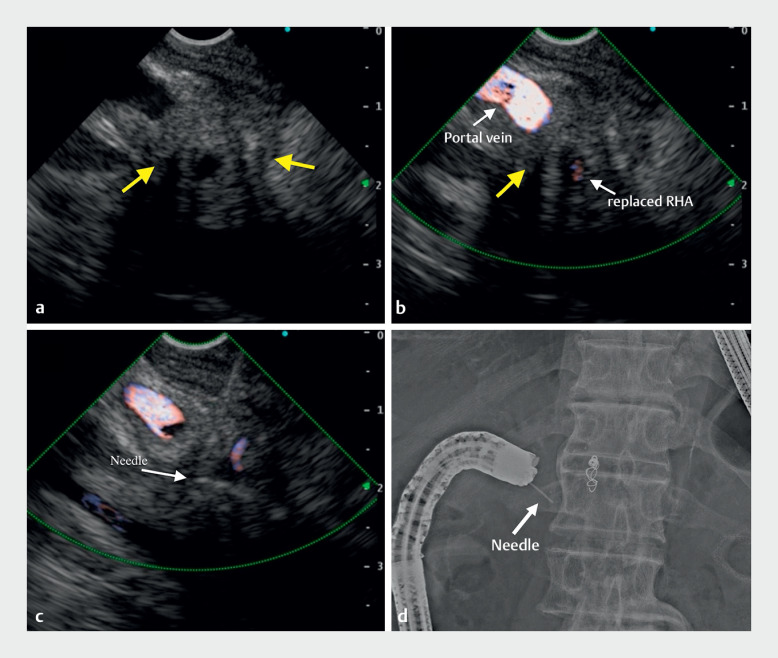
Endoscopic ultrasound-guided tissue acquisition. **a** EUS view of the hypoechoic mass (arrow) with B mode. **b** EUS view of the hypoechoic mass (arrow) using the color Doppler function. RHA, right hepatic artery. **c** Puncture of the mass under EUS guidance using a 22-gauge fine-needle biopsy needle. **d** Fluoroscopic image during EUS-TA.

**Fig. 5:**
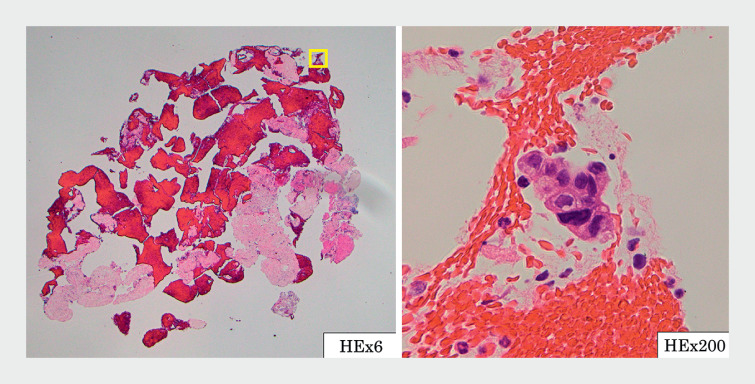
Histopathological appearance, revealing adenocarcinoma.

Endoscopic ultrasound-guided tissue acquisition successfully performed via the afferent limb using a forward-viewing echoendoscope in a patient with previous pancreaticoduodenectomy with modified Child’s reconstruction.Video 1

In cases of hilar lesions after pancreaticoduodenectomy with Child’s reconstruction, EUS-TA using an oblique-viewing echoendoscope is often difficult because the lesion is far away since it is approached transgastrically. Use of a forward-viewing echoendoscope may enable safe insertion into the afferent limb and EUS-TA with a short puncture distance 5 .

Endoscopy_UCTN_Code_TTT_1AS_2AD

## Notes

Endoscopy E-Videos https://eref.thieme.de/e-videos
*E-Videos* is an open access online section of the journal *Endoscopy* , reporting on interesting cases and new techniques in gastroenterological endoscopy. All papers include a high-quality video and are published with a Creative Commons CC-BY license. Endoscopy E-Videos qualify for HINARI discounts and waivers and eligibility is automatically checked during the submission process. We grant 100% waivers to articles whose corresponding authors are based in Group A countries and 50% waivers to those who are based in Group B countries as classified by Research4Life (see: https://www.research4life.org/access/eligibility/ ). This section has its own submission website at https://mc.manuscriptcentral.com/e-videos .
